# p300 suppresses leukemia development in NUP98-HOXD13 driven myelodysplastic syndrome

**DOI:** 10.18632/oncotarget.23402

**Published:** 2018-06-01

**Authors:** Na Man, Stephen D. Nimer

**Affiliations:** Sylvester Comprehensive Cancer Center and The Department of Biochemistry and Molecular Biology, Miller School of Medicine University of Miami, Miami, FL, USA

**Keywords:** myelodysplastic syndrome, acute myeloid leukemia, NUP98-HOXD13, lysine acetyltransferases, p300

Myelodysplastic syndromes (MDS) are clonal stem cell disorders that are characterized by ineffective hematopoiesis with bone marrow dysplasia, peripheral blood cytopenias and a predisposition to progress to acute myeloid leukemia (AML). Based on the identification of specific molecular lesions in MDS patients, including somatic mutations in a number of genes such as ASXL1, TET2, IDH1/2 and DNMT3A, several genetically modified MDS mouse models have been established [1]. Despite the rarity of the t(2;11)(q31;p15) in MDS, NUP98-HOXD13 (NHD13)-driven MDS mice display HOXA cluster gene activation, peripheral blood cytopenias and bone marrow dysplasia with impaired differentiation and increased apoptosis, which accurately reflects the clinical behavior of human MDS. We have used this model to study the roles of TP53, MSI2 and now the lysine acetyltransferases (KATs) EP300 and CREBBP in MDS.

KATs generally function as transcriptional co-activators by regulating gene expression and protein-protein interactions and modifying both histone and non-histone substrates. Inactivating mutations in the EP300 and CREBBP genes are found in a number of cancer types, including 3% of MDS patients and around 40% of patients with B-cell non-Hodgkin lymphoma (B-NHL), where they play a major pathogenetic role [2]. Low-level p300 expression is also found in B-lymphoma cell lines, accompanied by decreased histone H3 acetylation and acetylation-mediated dysregulation of a number of target genes [3]. While this evidence suggests a tumor suppressive role for p300/CBP, we previously showed that p300 acetylates AML1-ETO on lysine 43 and contributes to its leukemogenecity [4].

To explore the specific roles of the EP300 and CREBBP genes in MDS, we crossed p300 or CBP conditional knockout mice with NUP98-HOXD13 transgenic (NHD13tg) mice. In this model, loss of p300 significantly accelerated the onset of AML as all NHD13+, p300 null mice died from AML as early as 4 weeks after p300 deletion. In contrast, lack of CBP had no effect at all on the development of MDS, AML or bone marrow failure in the NHD13tg mouse model. This argues that the function of p300 and CBP is cell-context dependent in cancer cells, and despite similarities in structure and function, p300 and CBP can have divergent roles in pathogenesis. Mechanistically, we found that the deletion of p300 restored the hematopoietic stem and progenitor cell (HSPC) population in NHD13tg mice and promoted NHD13+ HSPC self-renewal, through increased symmetric self-renewing stem cell divisions. Again the absence of CBP had no such effect on NHD13+ HSPC biology, demonstrating the non-redundant roles of p300 and CBP. Moreover, a unique gene expression signature triggered by loss of p300 in NHD13-transformed HSPCs, but not normal HSPCs, included enhanced cytokine signaling and MAPK and JAK/STAT pathways, possibly conferring a growth advantage to the pre-leukemic blasts.

Our results indicate a tumor suppressor role for p300, blocking AML transformation in the NHD13-driven MDS (Figure 1) [5]. However, many mechanistic questions remain. As a histone acetyltransferase, loss of p300 may alter histone acetylation patterns globally or more specifically. Thus, comparing histone acetylation patterns in NHD13 transformed HSPCs (with or without p300) with the gene expression signature will help us better understand how p300 modifies the epigenetic landscape and controls the MDS-AML transition. p300 can also acetylate a wide range of non-histone proteins and altered modification of these substrates may also have profound cellular consequences. Exploring the network of p300 substrates in several defined MDS models will help uncover the mechanisms underlying the role of p300 in MDS. We are in the process of defining this role in MDS mouse models, based on commonly mutated genes, such as ASXL1, TET2 or DNMT3A.

**Figure 1:**
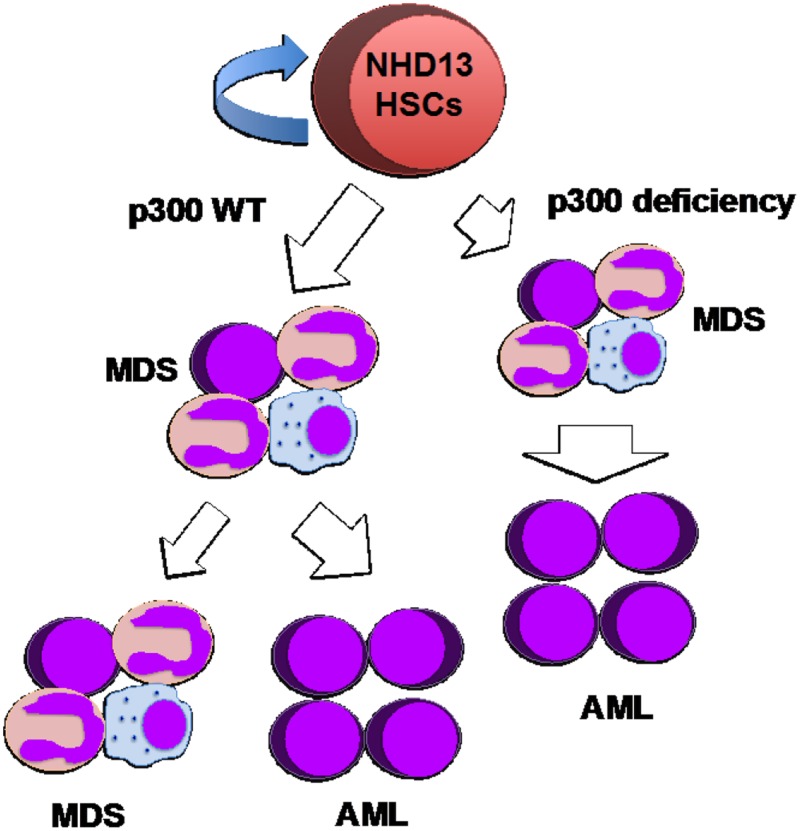
p300 functions as a tumor suppressor in NHD13-driven MDS.

While histone deacetylase inhibitors (HDACi) are being prescribed in the clinic (primarily for lymphoma patients), agents targeting KATs have not been systematically studied in solid cancers or hematopoietic malignancies and there are no FDA approved agents in this category. Therefore, there is an urgent need to advance the study of this group of anti-cancer agents. However, the context-dependent functions of KATs in hematologic malignancies suggest that caution be used evaluating agents targeting KATs. Pan KAT inhibitors have been shown to induce apoptosis and cell-cycle arrest in multiple AML cell lines and decrease the clonogenic growth of primary AML cells from patients, consistent with a pro-oncogenic role in various subtypes of AML [6]. However, our genetic study indicates that p300 can serve as a tumor suppressor gene in MDS, thus KAT’s inhibitor may not benefit certain cohorts of patients. On the other hand, specific pharmacological enhancers of p300 function are becoming available. The first p300 enhancer, N-(4-chloro-3-trifluoromethyl-phenyl)-2-ethoxy-6-pentadecyl-benzamide (CTPB) and its simplified analog CTB were shown to active p300 *in vitro*; recently, another p300 enhancer, suberoylanilide, was identified [7, 8]. Nonetheless, the efficacy of such drugs remains to be evaluated *in vivo*.

In summary, the identification and characterization of novel KAT enhancers, that have pharmacological characteristics suitable for clinical use, are anxiously awaited. Cells lacking or deficient in certain acetyltransferases may be more or less prone to being fully transformed. Defining how to best identify and exploit cancer cell dependency on KATs, or HDACs, will yield important mechanistic and therapeutic information.

## References

[1] Kwok B (2015). Blood.

[2] Pasqualucci L (2011). Nature.

[3] Haery L (2014). Mol Cancer.

[4] Wang L (2011). Science.

[5] Cheng G (2017). Leukemia.

[6] Giotopoulos G (2016). Oncogene.

[7] Balasubramanyam K (2003). J Biol Chem.

[8] Liu Y (2006). J Biol Chem.

